# The Use of Long-Read Sequencing Technologies in Infection Control: Horizontal Transfer of a *bla_CTX-M-27_* Containing lncFII Plasmid in a Patient Screening Sample

**DOI:** 10.3390/microorganisms10030491

**Published:** 2022-02-23

**Authors:** Vincent van Almsick, Franziska Schuler, Alexander Mellmann, Vera Schwierzeck

**Affiliations:** 1 Institute of Hygiene, University Hospital Münster, Robert-Koch-Straße 41, 48149 Münster, Germany; vincentfrederic.vanalmsick@ukmuenster.de (V.v.A.); alexander.mellmann@ukmuenster.de (A.M.); 2 Department of Cardiology I—Coronary and Peripheral Vascular Disease, Heart Failure, University Hospital Münster, 48149 Münster, Germany; 3 Institute for Medical Microbiology, University Hospital Münster, 48149 Münster, Germany; franziska.schuler@ukmuenster.de

**Keywords:** long-read sequencing, *bla_CTX-M-27_*, plasmid transfer

## Abstract

Plasmid transfer is one important mechanism how antimicrobial resistance can spread between different species, contributing to the rise of multidrug resistant bacteria (MDRB) worldwide. Here were present whole genome sequencing (WGS) data of two MDRB isolates, an *Escherichia coli* and a *Klebsiella quasipneumoniae,* which were isolated from a single patient. Detailed analysis of long-read sequencing data identified an identical F2:A-:B- lncFII plasmid containing *bla_CTX-M-27_* in both isolates, suggesting horizontal plasmid exchange between the two species. As the plasmid of the *E. coli* strain carried multiple copies of the resistance cassette, the genomic data correlated with the increased antimicrobial resistance (AMR) detected for this isolate. Our case report demonstrates how long-read sequencing data of MDRB can be used to investigate the role of plasmid mediate resistance in the healthcare setting and explain resistance phenotypes.

## 1. Introduction

The spread of multidrug resistant bacteria (MDRB) has become a serious threat for healthcare systems worldwide as antimicrobial resistance (AMR) limits treatment options even for life-threatening infections [1]. Horizontal gene transfer enables bacteria to easily share antimicrobial resistance genes (ARG) between various species by exchanging AMR plasmids [2,3,4]. Yet, little is known about the impact horizontal plasmid transfer has on the transmission and epidemiology of AMR in the hospital setting. One limiting factor is the use of short-read sequencing, as the technology has difficulties to distinguish if ARGs are located on the chromosome or on a plasmid. However, since long-read sequencing technology has been introduced into routine microbiology laboratories, this information has become more readily available [5,6] and can help in the detection and analyzation of ARG movement by plasmid conjugation and other mobile genetic elements [6].

In this article, we wanted to show how long-read sequencing data can be used to identify plasmid-mediated multidrug resistance (MDR) in a routine clinical sample. Our data shows evidence of a plasmid transfer between an *Escherichia coli* and a *Klebsiella quasipneumonia* isolate. In addition, detailed analysis of the MDR plasmid revealed an “artificial” multicopy of the AMR cassette, leading to a significantly higher minimum inhibitory concentration (MIC) against antibiotics.

## 2. Materials and Methods

### 2.1. Bacterial Strain Collection

Anorectal swab samples were collected as part of routine surveillance for multiresistant Gram negative rods. Samples were cultivated on chromID^®^ ESBL Agar (biomérieux, Marcy-l’Etoile, France), CHROMagar™ Acinetobacter (Mast Group, Paris, Frankreich), and Pseudomonas Cetrimide Agar (Thermo Fisher Scientific, Basingstoke, UK) and incubated at 36°C +/−1°C.

### 2.2. Identification of Bacteria and Antimicrobial Susceptibility Testing

Species identification was performed by matrix-assisted laser desorption/ionisation time-of-flight mass spectrometry (MALDI-TOF/MS) (Bruker, Bremen, Germany) with scores above 2.0. Antimicrobial susceptibility testing was performed using VITEK^®^ 2 (biomérieux, Marcy-l’Etoile, France) and Etest^®^ (biomérieux, Marcy-l’Etoile, France). MICs for antibiotics were interpreted according to the 2022 European Committee on Antimicrobial Susceptibility Testing (EUCAST, v.11.0) criteria.

### 2.3. Whole Genome Sequencing (WGS) and Data Analysis

Genomic DNA (gDNA) of bacterial isolates was extracted using the NEB Monarch Genomic Purification Kit (New England Biolabs, Ipswich, MA, USA). Both isolates were sequenced on a PacBio^®^ Sequel IIe system (Pacific Biosciences, Menlo Park, CA, USA) as described previously [7]. Next, raw sequences were assembled *de novo* and analyzed using the SMRT^®^ Link software suite v.9 with default parameters. Isolates were genotyped based on core genome multi-locus sequence typing (cgMLST) targets implemented in Ridom SeqSphere^+^ software version v.8.3.0 (Ridom GmbH, Münster, Germany) [8]. In addition, the multilocus sequence types (ST) were identified using the schemes for the respective species [9,10]. AMR genes and their location were determined using target gene sets for antimicrobial resistance based on the NCBI AMRFinderPlus [11]. To predict the plasmid replicon type the respective contigs were checked for complete circularization and uploaded to PlasmidFinder v.2.1 (24 January 2022: https://cge.cbs.dtu.dk/services/PlasmidFinder/) as well as pMLST 2.0 (24 January 2022: https://cge.cbs.dtu.dk/services/pMLST/) [12]. For annotation, we used the online Pipeline dfast (including HMM scan against TIGRFAM and RPSBLAST against COG database from NCBI) [13]. We performed BLAST analyses using BLASTn [14] and used BRIG Software (BLAST Ring Image Generator) [15] and progressive Mauve [16] for visualization.

## 3. Results

### 3.1. Species Identification and Antimicrobial Susceptibility Testing

During routine MDR screening upon intensive care unit (ICU) admission, two MDR isolates, an *E. coli* and a *K. quasipneumoniae*, were identified in a single patient. Growth on culture media was detected on chromID^®^ ESBL Agar after 24 h with two different morphotypes. Both isolates showed phenotypically decreased antimicrobial susceptibility to various antibiotics, including cephalosporins, beta-lactam/beta-lactamase inhibitors and fluoroquinolones and were suspected to be extended spectrum beta-lactamase (ESBL) producers (Table 1). Etest^®^ confirmed varying MICs for *E. coli* (Cefotaxime > 32 mg/L, Ceftazdime 12 mg/L, Ciprofloxacin 3 mg/L) and *K.* *quasipneumoniae* (Cefotaxime > 32 mg/L, Ceftazidime 1.5 mg/L, Ciprofloxacin 0.38 mg/L).

### 3.2. Detection of ARG by WGS Data Analysis

As all MDR isolates are subjected to WGS as part of the routine hospital surveillance at the University Hospital Münster, both isolates were sequenced using long-read technology. Data analysis identified the *E. coli* isolate as multilocus sequence type (ST) 1722 and the *K. quasipneumoniae* isolate as ST138. Further data analysis for ARG identified *bla_CTX-M-27_* in both isolates as well as numerous ARG such as *bla_LAP-2_* and *qnrS1*. In addition, the *K. quasipneumoniae* carried the *dfrA26* resistance gene matching phenotypic resistance to trimethoprim (Table 1 and Table 2).

### 3.3. Characterisation and Comparison of MDR Plasmids

**Figure 1:**
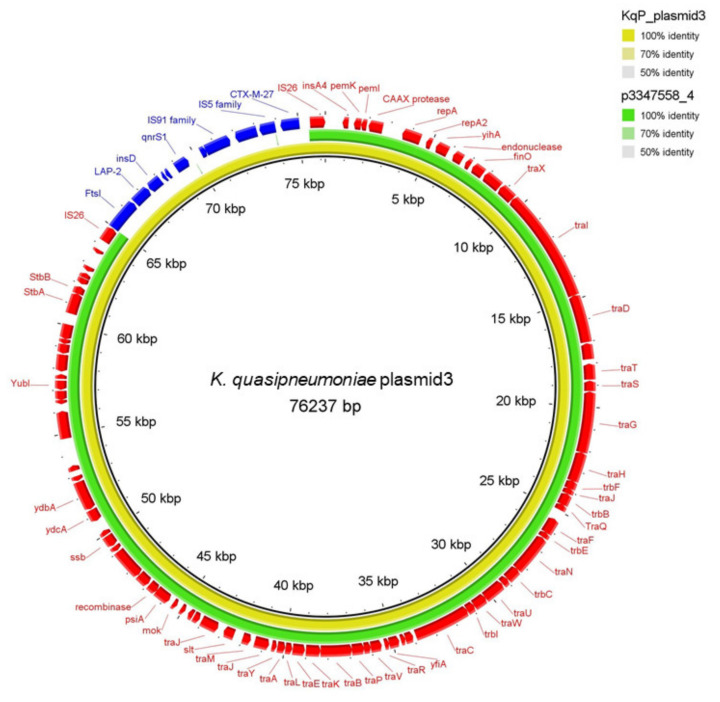
Comparison of the *K. quasipneumoniae* plasmid and the reference plasmid p3347558_4 using BRIG. The outer Ring shows the annotated sequences by dfast: The AMG cassette is colored in blue, all other proteins are colored in red.

**Figure 2:**
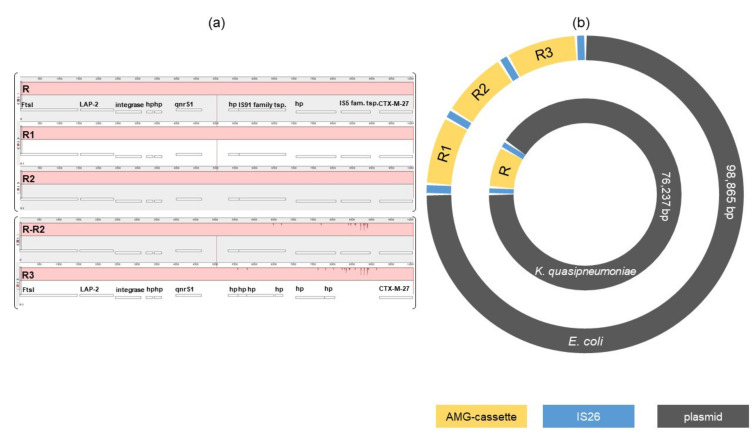
(**a**): progessiveMauve alignment of the AMG-cassettes: R = AMG-cassette of the *K. quasipneumoniae* plasmid; R1-3 = AMG-cassettes of the *E. coli* plasmid; FtsI = peptidoglycan glycosyltransferase FtsI; LAP-2 = bla*_LAP-2_*; hp = hypotetical protein; qnrS1 = quinolone resistance protein *qnrS1*; tsp. = transpoase; CTX-M-27 = bla*_CTX-M-27_*. IS91 & IS5 family tsp. lost in R3 by frameshift because of insertions and deletions. (**b**): schematic blueprint of the two plasmids.

Next, we investigated if ESBL resistance genes were located on a plasmid or encoded on the chromosome. The web-tools PlasmidFinder predicted a IncFII plasmid and pMLST the FAB formula F2:A-:B for the contig that are carrying the *bla_CTX-M-27_* alongside several ARG for both isolates. The plasmid length was 98,865 bp and 76,237 bp in the *E. coli* and *K. quasipneumoniae* isolates, respectively. The *dfrA26* gene detected in the *K. quasipneumoniae* on the other hand was located on another plasmid. Both DNA sequences were annotated and showed 100% identical encoded proteins including a relaxome formed by TraI relaxase, TraM and TraY proteins and a type IV secretion system needed for self-conjugative plasmids [13,17] as well as the IncFII replicon (Figure 1). Visualization of the plasmids by BRIG using BLASTn alignment confirmed a 100% sequence match. The difference in total length of the plasmids is caused by the additional two copies of the AMR cassette (Figure 2b). The comparison using progressiveMauve alignment of the AMG-cassettes showed 100% identity of the *K. quasipneumoniae* AMG-cassette compared to the first two copies in the *E. coli* plasmid (Figure 2a). The third copy has some deletions and insertions leading to a frameshift causing the loss of the IS91 family transposase and the IS5 family transposase (Figure 2a). An online BLASTn search came up with several similar plasmids but without the AMR cassette. The nearest ancestor plasmid, calculated by BLASTn (Percent Query Coverage: 86%; Percent Identity: 99.95%, for BRIG visualisation see Figure 1), was isolated in 2020 in Switzerland (NCBI GenBank accession no. CP071077.1, plasmid p3347558_4) [18]. Overall, these results strongly suggest horizontal plasmid transfer has occurred between these two strains at one point in the past. Interestingly, the number of copies of the *bla_CTX-M-27_* resistance cassette correlated with the increased MIC of cephalosporins and fluoroquinolones detected in the *E. coli* isolate compared to the *K. quasipneumoniae* isolate (Table 2 and Figure 2).

## 4. Discussion

Many reports support the view that plasmid transfer is a relevant mechanism how AMG spread within the hospital setting [19,20,21,22]. However, investigating plasmid transfer based on short-read sequencing data is challenging. Here we present data illustrating how long-read sequencing can be used as a high-resolution tool to monitor plasmids as part of the molecular surveillance of MDR isolates in the hospital setting. Our analysis strongly suggests an inter-species transfer of a self-conjugative F2:A-:B- plasmid between an *E. coli* and a *K. quasipneumoniae* isolate in vivo. While the exact time point and source of the plasmid transfer remains unclear, our observation demonstrates that this plasmid is capable to adapt to two different enterobacterial species. In fact, the plasmid described might possess a rather broad host range due to its ability to self-conjugate. Additionally, we could correlate the resistance phenotype of the MDR isolates with their genotype as sequencing data revealed three copies of the AMG cassette, explaining the increased MIC for cephalosporins and fluoroquinolones observed in the *E. coli* isolate. This example highlights the impact of duplication of AMG cassettes during or through plasmidal conjugations. The mechanisms how duplication of resistance cassettes occur in vivo and how this affects resistance phenotypes are not completely understood yet and will need further investigation as so far only few cases of clinical isolates have been described [23]. Medical records showed that the patient colonized with these MDR isolates has received prolonged treatment with cotrimoxazol/sultamicillin over several weeks because of recurred urinary tract infections (URTIs) prescribed by his general practitioner as well as piperacillin/tazobactam and meropenem twice while being admitted as inpatient in hospital. Fluoroquinolones were not used in the patient’s treatment. However, we must assume that even under selective pressure of a single antibiotic, the entire gene cassette is transferred and the ARG of the other antibiotic classes are co-selected, regardless of the antibiotic chosen previously. In the case of the isolate described this caused increased MIC of fluoroquinolones without using them in the patient’s treatment.

## 5. Conclusions

Taken together our data shows how analyzing long-read WGS data of routine MDR isolates can give insight into the transfer and function of plasmids in the hospital setting and can help to correlate genotype with resistance phenotype. Plasmid transfer and interaction with other mobile genetic elements in vivo are not completely understood yet but long-read WGS is a valuable tool to address this question and analyze MDR isolates in greater detail even in a routine laboratory setting.

## Figures and Tables

**Table 1:** Antibiotic susceptibility of MDRB isolates. MIC = minimum inhibitory concentration; EUCAST = European Committee on Antimicrobial Susceptibility Testing; R = resistant; S = susceptible.

Antibiotics	Classification by EUCAST		MIC (mg/L)	
	***E.coli* (1)**	*K. quasipneumoniae* (2)	1	2
Ampicillin	R	R	>=32	>=32
Amoxicillin	R	R	-	-
Amoxicillin/Sulbactam	R	R	16	16
Piperacillin/Tazobactam	S	S	<=4	<=4
Cefuroxime	R	R	>=64	>=64
Cefotaxime	R	R	>32	>32
Cefpodoxime	R	R	>=8	>=8
**Ceftazidime**	**R**	**I**	**12**	**1.5**
Ertapenem	S	S	<=0.5	<=0.5
Imipenem	S	S	<=0.25	<=0.25
Meropenem	S	S	<=0.25	<=0 25
Gentamicin	R	R	<=1	<=1
**Ciprofloxacin**	**R**	**R**	**3**	**0.38**
Levofloxacin	R	R	-	-
**Moxifloxacin**	**R**	**R**	**>=8**	**2**
Tigecyclin	S		<=0.5	-
**Trimethoprim/Sulfa**	**S**	**R**	**<=20**	**>=320**

**Table 2:** ARGs identified by AMRFinderPlus.

Class	Gene Symbol	Aligned Overlap (%)	Location
*K. quasipneumoniae*			
BETA-LACTAM	*bla_OKP-B-2_*	100	KqP_chromosome
FOSFOMYCIN	*fosA*	100	KqP_chromosome
PHENICOL/QUINOLONE	*oqxB*	100	KqP_chromosome
PHENICOL/QUINOLONE	*oqxA*	100	KqP_chromosome
BETA-LACTAM	*bla_TEM-1_*	100	KqP_plasmid2
SULFONAMIDE	*sul2*	100	KqP_plasmid2
TETRACYCLINE	*tet(D)*	100	KqP_plasmid2
**TRIMETHOPRIM**	***dfrA26***	**100**	**KqP_plasmid2**
**BETA-LACTAM**	***bla_LAP-2_***	**100**	**KqP_plasmid3**
**BETA-LACTAM**	***bla_CTX-M-27_***	**100**	**KqP_plasmid3**
**QUINOLONE**	***qnrS1***	**100**	**KqP_plasmid3**
*E. coli*			
FOSFOMYCIN	*uhpT_E350Q*	100	EC_chromosome
FOSMIDOMYCIN	*cyaA_S352T*	100	EC_chromosome
QUINOLONE	*gyrA_S83L*	99.6	EC_chromosome
AMINOGLYCOSIDE	*aph(3’)-Ia*	100	EC_plasmid1
AMINOGLYCOSIDE	*aph(6)-Id*	100	EC_plasmid1
AMINOGLYCOSIDE	*aph(3’’)-Ib*	100	EC_plasmid1
BETA-LACTAM	*bla_TEM-1_*	100	EC_plasmid1
SULFONAMIDE	*sul2*	100	EC_plasmid1
TETRACYCLINE	*tet(B)*	100	EC_plasmid1
**BETA-LACTAM**	***bla_LAP-2_***	**100**	**EC_plasmid2**
**BETA-LACTAM**	***bla_LAP-2_***	**100**	**EC_plasmid2**
**BETA-LACTAM**	***bla_LAP-2_***	**100**	**EC_plasmid2**
**BETA-LACTAM**	***bla_CTX-M-27_***	**100**	**EC_plasmid2**
**BETA-LACTAM**	***bla_CTX-M-27_***	**100**	**EC_plasmid2**
**BETA-LACTAM**	***bla_CTX-M-27_***	**100**	**EC_plasmid2**
**QUINOLONE**	***qnrS1***	**100**	**EC_plasmid2**
**QUINOLONE**	***qnrS1***	**100**	**EC_plasmid2**
**QUINOLONE**	***qnrS1***	**100**	**EC_plasmid2**

## Data Availability

The sequence information presented in this study has been deposited under NCBI BioProject accession PRJNA802079.
